# Viroid Replication: Rolling-Circles, Enzymes and Ribozymes

**DOI:** 10.3390/v1020317

**Published:** 2009-09-14

**Authors:** Ricardo Flores, María-Eugenia Gas, Diego Molina-Serrano, María-Ángeles Nohales, Alberto Carbonell, Selma Gago, Marcos De la Peña, José-Antonio Daròs

**Affiliations:** Instituto de Biología Molecular y Celular de Plantas (UPV-CSIC), Avenida de los Naranjos s/n, 46022 Valencia, Spain; E-Mails: mgas@ibmcp.upv.es (M.-E.G.); dmolina@ibmcp.upv.es (D.M.-S.); manozaf@ibmcp.upv.es (M.-A.N.); alcarol@ibmcp.upv.es (A.C.); selmag@ibmcp.upv.es (S.G.); rivero@ibmcp.upv.es (M.D-P.)

**Keywords:** viroids, catalytic RNAs, hammerhead ribozymes

## Abstract

Viroids, due to their small size and lack of protein-coding capacity, must rely essentially on their hosts for replication. Intriguingly, viroids have evolved the ability to replicate in two cellular organella, the nucleus (family *Pospiviroidae)* and the chloroplast (family *Avsunviroidae).* Viroid replication proceeds through an RNA-based rolling-circle mechanism with three steps that, with some variations, operate in both polarity strands: i) synthesis of longer-than-unit strands catalyzed by either the nuclear RNA polymerase II or a nuclear-encoded chloroplastic RNA polymerase, in both instances redirected to transcribe RNA templates, ii) cleavage to unit-length, which in the family *Avsunviroidae* is mediated by hammerhead ribozymes embedded in both polarity strands, while in the family *Pospiviroidae* the oligomeric RNAs provide the proper conformation but not the catalytic activity, and iii) circularization. The host RNA polymerases, most likely assisted by additional host proteins, start transcription from specific sites, thus implying the existence of viroid promoters. Cleavage and ligation in the family *Pospiviroidae* is probably catalyzed by an RNase III-like enzyme and an RNA ligase able to circularize the resulting 5′ and 3′ termini. Whether a chloroplastic RNA ligase mediates circularization in the family *Avsunviroidae,* or this reaction is autocatalytic, remains an open issue.

## 1. Introduction

Perhaps the most striking aspect of viroids from a functional perspective is that, in contrast to viruses, they lack protein-coding ability [1,2]. This feature directly linked to their structural peculiarities —viroids are RNAs of 246–301 nt that would limit the size of their potential translation products and, additionally, with a circular structure that would impede translation by a scanning mechanism starting from the 5′ terminus— has deep consequences in the context of replication. While RNA viruses encode subunits of the enzymatic complex (RNA replicase) that catalyzes initiation and elongation of viral RNA strands, viroids must rely for this replication step on pre-existing host RNA polymerases [2–7]. In principle, the best candidates would be RNA-dependent RNA polymerases, whose existence in plants has been known for a long time [8,9]. However, viroids do not use these enzymes for their replication, but DNA-dependent RNA polymerases redirected to accept RNA templates (see below). Why and how viroids have evolved this capacity is one of the most intriguing issues awaiting an answer. Additionally, some viroids have evolved a second outstanding capacity: they are catalytic RNAs “encoding” in their strands hammerhead ribozymes that play a crucial role in replication. Therefore, studies on how viroids replicate have revealed aspects of RNA metabolism that were totally unanticipated, some of which might also play a role in their hosts. Illustrating this point, naturally-encoded hammerhead ribozymes have been discovered in the genome of *Arabidopsis thaliana* [10] and mouse [11]; the latter, embedded in a messenger RNA, is able to reduce protein expression *in vivo*.

## 2. An overview of viroid singularity and diversity

**Figure 1.**
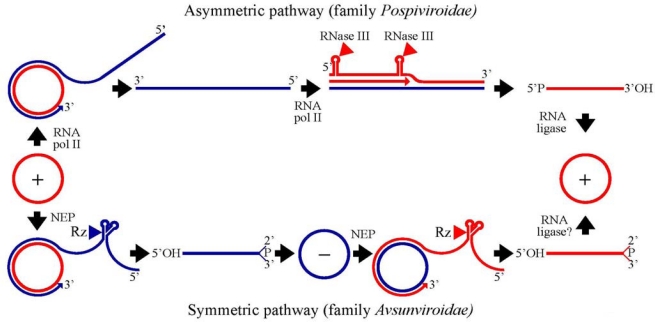
Rolling-circle replication mechanism with the two alternative pathways followed by members of the family *Pospivirodae* (asymmetric) and *Avsunviroidae* (symmetric) [15,16,46]. Red and blue lines refer to (+) and (−) strands, respectively. Arrowheads mark the cleavage sites of a host RNase III or a hammerhead ribozymes (Rz), and the resulting terminal groups are indicated. Elongation of RNA strands is catalyzed by the nuclear RNA polymerase II (RNA pol II) or the nuclear-encoded chloroplastic RNA polymerase (NEP).

Replication of viroids is very much dependent of their intrinsic properties and of the different subcellular organella in which this process occurs. Sequence and structural analyses of the approximately 30 viroids characterized so far have revealed that they can be clustered into two families: i) *Pospiviroidae,* type species *Potato spindle tuber viroid* (PSTVd), the members of which exhibit a central conserved region (CCR) and most adopt a rod-like secondary struture, and ii) *Avsunviroidae,* type species *Avocado sunblotch viroid* (ASBVd), whose members lack a CCR but have the ability to form hammerhead ribozymes in both polarity strands, and fold into quasi-rod-like or clearly branched secondary structures. Allocation of viroid species into genera is based on additional criteria. The two families also differ regarding their functional properties: the available evidence indicates that while members of the family *Pospiviroidae* have a nuclear replication (and accumulation), those of the family *Avsunviroidae* replicate (and accumulate) in the chloroplast [4,12]. These very different subcellular sites have deep implications on the enzymology underlying viroid replication. Viroids replicate through an RNA-based rolling-circle mechanism with three steps that, with some variations, operate in the strands of both polarities: i) synthesis of longer-than-unit strands catalyzed by a host nuclear or chloroplastic RNA polymerase that reiteratively transcribes the initial circular template, ii) processing to unit-length, which remarkably is mediated by hammerhead ribozymes in the family *Avsunviroidae*, and iii) circularization resulting from the action of an RNA ligase or occuring autocatalytically (Figure 1).

Viroids share structural and functional similarities with the so-called viroid-like satellite RNAs (small, circular, non-protein-coding genomes that replicate through a rolling-circle mechanism involving ribozymes) but, in contrast to viroids, viroid-like satellite RNAs depend for their replication on a helper virus [13]. The RNA of *Human hepatitis delta virus* also bears some resemblance with viroids (circularity and replication through a rolling-circle mechanism mediated by ribozymes), although it has a larger size and encodes a protein in its antigenomic polarity [14]. How viroids, with such a tiny genome, are able to promote their own replication? Here we will review hypotheses and results on this issue, putting special emphasis on those most recent that have challenged some longheld tenets.

## 3. Family *Pospiviroidae:* an asymmetric rolling-circle mechanism with circular and linear templates catalyzed by host enzymes

The basis for this model were founded by early experiments performed with PSTVd and, to a lesser extent, with other members of the same genus *(Pospiviroid)* [4,15,16]. Replication starts when the incoming monomeric circular *(mc)* RNA —to this most abundant species *in vivo* is assigned the (+) polarity by convention— is reiteratively transcribed into oligomeric (−) RNAs that in turn serve as templates for synthesis of oligomeric (+) RNAs. These latter transcripts are then cleaved into the monomeric linear *(ml)* form and ligated to the mature *mc* (+) RNA, the final product of the cycle (Figure 1). This mechanism, which is termed asymmetric because the rolling-circle only operates for synthesis of the oligomeric (−) RNAs, is catalyzed by host enzymes that recognize specific RNA motifs. Assuming that the loops and bulges (flanked by double-stranded helices) predicted in the PSTVd secondary structure are functional motifs that regulate replication in single cells or systemic trafficking *in planta,* a recent genome-wide mutational analysis has identified multiple loops/bulges essential or important for each of these biological processes [17]. The resulting genomic map indicates that motifs critical for replication are concentrated in the CCR and in the left terminal domain. This view is supported by additional independent data (see below).

### 3.1. Initiation and elongation: involvement of RNA polymerase II

Two lines of evidence support that the enzyme catalyzing elongation of PSTVd and closely-related viroids is RNA polymerase II: i) nanomolar concentrations of the fungal toxin α-amanitin that typically inhibit this enzyme also block *in vivo* and *in vitro* transcription of (+) and (−) viroid strands, and ii) viroid (+) and (−) strands have been retrieved with a monoclonal antibody against the carboxyterminal domain of the largest subunit of RNA polymerase II [4].

Regarding the transcription initiation sites, this question has been recently tackled by priming a nuclear extract from a non-infected cell culture of the host plant *Solanum tuberosum* with the PSTVd *mc* (+) RNA. Following purification by affinity chromatography, analysis by primer extension of the (−) strands synthesized *de novo* revealed a single start site located in the hairpin loop of the left terminal domain of the rod-like secondary structure proposed for PSTVd *mc* (+) RNA [18]. Further analyses *in planta* of site-directed mutants are consistent with this hairpin loop being involved in infectivity and perhaps replication [18], as also is a genome-wide mutational analysis [17]. However, additional studies are needed to confirm this site as well as to identify the transcription initiation site of (+) strands, which remains unknown. The possibility exists that, like other RNA polymerase II transcripts, the 5′ termini of (+) and (−) RNAs of nuclear viroids could be capped *in vivo,* a signature that would mark unambiguously their initiation sites.

### 3.2. Cleavage of oligomeric (+) strands is most likely mediated by a member of the RNase III family

Early studies implicated the CCR in processing of the oligomeric (+) strands of the family *Pospiviroidae* either through hairpin I, a metastable motif that can be formed by the upper CCR strand and flanking nucleotides during thermal denaturation [19], or through a thermodynamically stable double-stranded structure that can be alternatively assumed by the same sequences in oligomeric RNAs. Data supporting this view include infectivity bioassays with different PSTVd DNA and RNA constructs [20], with longer-than-unit clones of *Hop stunt viroid* (HSVd) [21], and with constructs of *Citrus exocortis viroid* (CEVd) containing sequence repetitions and point mutations in the upper CCR strand [22]. A critical reassessment of these data led to a model involving the double-stranded structure in cleavage, although the model did not predict the mechanism of cleavage-ligation or specify the exact processing site [23]. On the other hand, *in vitro* and thermodynamic analyses of the products obtained by incubating a potato nuclear extract with a full-length PSTVd RNA containing a 17-nt repeat of the upper CCR strand led to propose that cleavage of (+) strands is driven by a multibranched structure with a hairpin —different from hairpin I— capped by a GAAA tetraloop conserved in members of the genus *Pospiviroid.* This multibranched structure subsequently switches to an extended conformation with a loop E (see below) that promotes ligation [24]. Yet, the processing complex formed *in vitro* may not mimic the corresponding complex *in vivo*. Moreover, other members of the family *Pospiviroidae* cannot form the hairpin capped by a GAAA tetraloop [4], and alternative processing sites in the lower CCR strand, or outside this region, have been observed for several members of this family [25].

This question has been recently examined *in vivo* [26], using a system based in transgenic *A. thaliana* expressing dimeric (+) transcripts, *dt* (+) RNAs, of CEVd, HSVd, and *Apple scar skin viroid* (ASSVd) [27] of the genera *Pospiviroid, Hostuviroid,* and *Apscaviroid,* respectively, within the family *Pospiviroidae* [4]. This system circumvents most of these limitations. It is an *in vivo* system in which processing is correct: transgenically expressed dimeric transcripts of typical members of the family *Pospiviroidae* are cleaved to the *ml* forms and then ligated to the infectious *mc* RNAs, whereas the complementary *dt* (−) RNAs are not [26,27], thus reproducing the situation observed in typical hosts. However, in contrast to typical hosts in which turnover of the longer-than-unit (+) replicative intermediates is difficult to follow because of their low accumulation and diverse size, the *A. thaliana-*based system provides a constant supply of a size-specific replicative-like intermediate that can be easily quantified as well as its processing products. Moreover, the *ml* and *mc* (+) RNAs can be assumed to come essentially from processing of the transgenically expressed *dt* (+) RNA. Therefore, the effects of specific mutations in the primary transcript on cleavage and ligation can be evaluated — regardless of whether the resulting products are infectious or not — and it is even possible to identify mutations affecting only ligation (see below).

**Figure 2.**
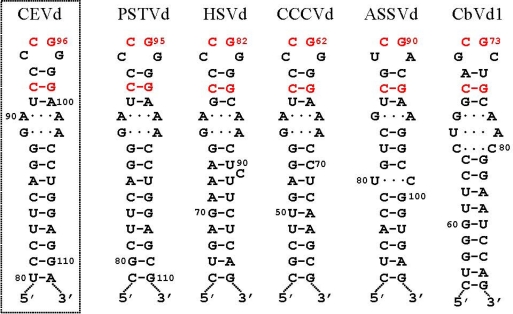
Hairpin I structures of the five type species of the family *Pospiviroidae.* This structural element is formed by the upper CCR strand and flanking nucleotides of the type members of the five genera composing the family *Pospiviroidae:* PSTVd, HSVd, CCCVd *(Coconut cadang-cadang viroid),* ASSVd*,* and CbVd1 *(Coleus blumei viroid 1)* [25,26]. Red fonts indicate conserved nucleotides in structurally similar positions. Continuous and broken lines represent Watson-Crick and non-canonical base pairs, respectively [28]. Notice that the variability preserves the overall structure of hairpin I, including the terminal palindromic tetraloop, the adjacent 3-bp stem, and the long stem. Left inset, hairpin I of the CEVd variant used to transform *A. thaliana* [26]; notice two covariations with respect to PSTVd at the basis of the long stem. Reprinted with permission from [26].

**Figure 3.**
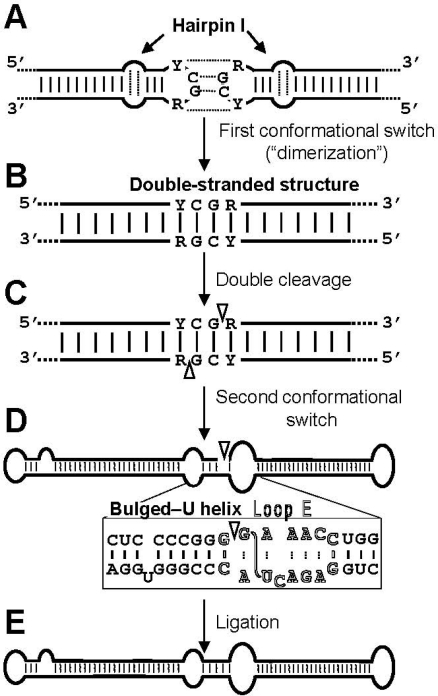
Model for processing *in vivo* of the oligomeric (+) replicative intermediates of the family *Pospiviroidae* that involves a kissing loop interaction between the palindromic tetraloops of two consecutive hairpin I motifs **(A)**, with their stems forming subsequently a longer interstrand duplex **(B)**. This double-stranded structure is the substrate for cleavage at specific positions in both strands **(C)**. Following a second conformational switch the resulting unit-length strands adopt the extended rod-like structure with loop E (in outlined fonts) and the adjacent bulged-U helix **(D)**, which is the substrate for ligation **(E)**. R and Y refer to purines and pyrimidines, respectively, the S-shaped line denotes the UV-induced cross-link, and white arrowheads mark the cleavage sites in the double-stranded structure and the ligation site in the extended conformation. Reprinted with permission from [26].

Results with the *A. thaliana-*based system have mapped the cleavage site of CEVd (+) strands at the upper CCR strand [26], in a position equivalent to that inferred for PSTVd with an *in vitro* system [24]. However, the RNA motif directing cleavage *in vivo* does not seem to be the GAAA-capped hairpin proposed previously [24], but the hairpin I/double-stranded structure [26]. The first argument supporting this view is that whereas the cleavage sites of HSVd and ASSVd (+) strands also map at equivalent positions in a similar hairpin I/double-stranded structure, these viroids cannot form the GAAA-capped hairpin. In contrast, examination of the hairpin I/double-stranded structure reveals some appealing features. Hairpin I is composed by a tetraloop, a 3-bp stem, an internal symmetric loop of 1–3 nt in each strand that presumably interact by non-Watson-Crick base pairs [28], and a 9–10-bp stem that can be interrupted by a 1-nt symmetric or asymmetric internal loop [22,25] (Figure 2). Remarkably, these structural features are conserved in the type species of the five genera composing the family *Pospiviroidae* and additionally: i) the capping tetraloop is palindromic itself, and ii) the two central positions of the tetraloop and the central base pair of the 3-bp stem are phylogenetically conserved (Figure 2) [25]. As a consequence, a long double-stranded structure with a GC-rich central region of 10 bp containing the cleavage sites can be alternatively assumed by the same sequences in a di- or oligomeric RNA (Figure 3). The second argument supporting the hairpin I/double-stranded structure as the RNA motif directing cleavage derives from the effects on this reaction of different CEVd mutants expressed transgenically in *A. thaliana* affecting differentially this motif versus the GAAA-capped hairpin [26].

Altogether, the results obtained with the *A. thaliana-*based system and with some experimental hosts [26] indicate that the substrate for cleavage *in vivo* of all members of the family *Pospiviroidae* is the double-stranded structure proposed previously [23], with hairpin I playing a role in promoting the adoption of this structure (see below). Moreover, the cleavage sites in the double-stranded structure leave two 3′-protruding nucleotides in each strand (Figure 3), the characteristic signature of RNase III enzymes [29,30]. The participation of an enzyme of this class, of which there are at least seven in *A. thaliana* [31], is consistent with the nuclear location of some of them, which additionally have preference for substrates with a strong secondary structure resembling that of viroids. Going one step further, if an RNase III indeed catalyzes cleavage of the oligomeric (+) RNAs of the family *Pospiviroidae,* the resulting products should have 5′-phosphomonoester and free 3′-hydroxyl termini. Characterization of the *ml* (+) RNAs from *A. thaliana* transgenically expressing *dt* CEVd (+) RNAs has shown that this is actually the case [32]. The adoption *in vivo* of the double-stranded structure with a GC-rich central region containing the cleavage sites could be promoted by hairpin I because prior work with PSTVd has mapped a dimerization domain at this hairpin [28]. This situation resembles that observed previously in retroviruses in which dimerization, a critical step of their infectious cycle, is mediated by a hairpin with a palindromic loop that can dimerize via a kissing loop interaction [33]. During transcription of oligomeric (+) RNAs of the family *Pospiviroidae,* a kissing loop interaction between the palindromic tetraloops of two consecutive hairpin I motifs might similarly start intramolecular dimerization, with their stems then forming a longer interstrand duplex [32] (Figure 3).

### 3.3. Ligation of monomeric linear (+) strands: presumable participation of a novel RNA ligase

Initial *in vitro* assays showed that incubation with wheat germ RNA ligase converts the monomeric linear (+) PSTVd RNA isolated from infected tissue into its *bona fide* circular counterpart, suggesting that the reaction might be mediated by an enzyme of this class [34], which demands 5′-hydroxyl and 2′,3′-cyclic phosphodiester termini [35]. Although subsequent experiments with the fungal RNase T1 revealed that it can process *in vitro* linear oligomeric (+) PSTVd RNAs into infectious monomeric circular RNA [36], it is unlikely that an RNAse could catalyze *in vivo* both cleavage and ligation. Moving into a more physiological context, incubation with a potato nuclear extract of a monomeric (+) PSTVd RNA with a short repeat of the CCR upper strand produced the infectious monomeric circular form, leading to the proposal that the enzymatic cleavage and ligation of the PSTVd (+) strand is driven by a switch from a branched structure containing a GAAA-capped hairpin (see above) to an extended conformation with an E loop [24]. Loop E, a UV-sensitive motif of RNA tertiary structure that is conserved in PSTVd and members of its genus [4] and exists *in vitro* [37] and *in vivo* [38,39], has also been involved in host specificity [40], pathogenesis [41], and transcription [42]. A structural model based on isostericity matrix and mutagenic analyses has been derived recently for PSTVd loop E [42]; this model can be extended to other closely-related viroids like CEVd.

With the aim of circumventing the intrinsic limitations of *in vitro* approaches, the most important of which is to what extent results from these approaches can be extrapolated to the *in vivo* situation, the *A. thaliana-*based system [27] described in the previous section for studying *in vivo* cleavage of the oligomeric (+) strands has also been used to examine the requisites for proper ligation of the resulting *ml* (+) RNAs. Data obtained with this system indicate that the substrate for this reaction in the genus *Pospiviroid* is the extended conformation containing loop E [26] (Figure 3). Therefore, whereas cleavage is only dependent on the upper CCR strand and flanking nucleotides, ligation is dependent on nucleotides of both CCR strands that encompass those of loop E and others adjacent. Because within the family *Pospiviroidae* loop E is only formed in the genera *Pospiviroid* and *Cocadviroid,* other genera of this family must have alternative motifs playing a similar role in ligation. Potential candidates are the extended conformation of the CCR with a bulged-U helix conserved in all members of the genera *Pospiviroid, Hostuviroid* and *Cocadviroid* (Figure 3D), and similar structures in the other genera of this family. More recent data, obtained by rapid amplification of 5′ and 3′ cDNA ends and *in vitro* ligation assays with the T4 RNA ligase 1 and *A. thaliana* tRNA ligase, have shown that the *ml* CEVd (+) RNA resulting from cleavage of a dimeric transcript transgenically expressed in *A. thaliana* indeed contains the 5′-phosphomonoester and 3′-hydroxyl termini expected from cleavage mediated by an RNase III [32]. Therefore, unless the termini are later modified, these results suggest the existence of a second plant RNA ligase that would mediate joining of the same 5′-P and 3′-OH termini as those required by the T4 RNA ligases 1 and 2 [43,44].

## 4. Family *Avsunviroidae:* a symmetric rolling-circle mechanism with circular RNA templates catalyzed by host enzymes and viroid ribozymes

This model is founded in results obtained with ASBVd [4,12,45,46], additionally supported with data generated with the other members of the family: *Peach latent mosaic viroid* (PLMVd) [47], *Chrysanthemum chlorotic mottle viroid* (CChMVd) [48] and *Eggpant latent viroid* (ELVd) [49]. In this family, the oligomeric (−) RNAs produced in the first rolling-circle are cleaved and ligated into the *mc* (−) form, then serving as template for a second rolling-circle leading to the oligomeric (+) RNA intermediates that are processed into the *mc* (+) RNA. Therefore, the second half of the cycle is symmetric with respect to the first. Most importantly, cleavage of the oligomeric RNA intermediates is autocatalytic and mediated by hammerhead ribozymes embedded in both polarity strands [50].

### 4.1. Initiation and elongation: involvement of a nuclear-encoded chloroplastic RNA polymerase

Two different RNA polymerases have been reported in plastids, the plastid-encoded polymerase (PEP) with a multisubunit structure similar to the *Escherichia coli* enzyme, and the single-unit nuclear-encoded polymerase (NEP) resembling phage RNA polymerases. The available evidence supports the involvement of the second in replication of chloroplastic viroids. First, adding micromolar concentrations of the toxin tagetin to chloroplastic preparations from ASBVd-infected leaves prevents *in vitro* transcription of representative chloroplastic genes without essentially affecting synthesis of ASBVd strands [51]. And second, accumulation of a typical NEP transcript and of PLMVd strands is particularly high in areas displaying peach calico (an albinism induced by specific PLMVd variants), in which development of proplastids into chloroplasts and processing of chloroplastic rRNA precursors (and translation of plastid-encoded proteins like PEP) is impaired [52]. These latter data also suggest that other chloroplastic proteins mediating replication (see below) are also nuclearencoded.

The question of the transcription initiation sites has been undertaken by *in vitro* capping with [α ^32^P]-GTP and guanylyl-transferase, which specifically labels the free 5′-triphosphate group characteristic of chloroplastic primary transcripts. This approach, combined with RNase protection assays, has identified the initiation sites of ASBVd *ml* (+) and (−) RNAs isolated from infected avocado at similar (A+U)-rich terminal loops in their predicted quasi-rod-like secondary structures [53]. The same methodology, but combined with RNA ligase-mediated rapid amplification of cDNA ends to enhance its sensitivity, has mapped the initiation sites of PLMVd *ml* (+) and (−) RNAs isolated from infected peach at similar double-stranded motifs of 6 to 7 bp that also contain the conserved GUC triplet preceding the self-cleavage site in both polarity strands; within their predicted branched secondary structures, the motifs are located at the base of similar long hairpins that presumably contain the NEP promoters [54]. These PLMVd initiation sites, which have been later confirmed [55], are different from those proposed previously on the basis of primer-extensions of the 5′ termini of PLMVd subgenomic RNAs isolated from infected peach, and of *in vitro* transcriptions with truncated PLMVd RNAs and the RNA polymerase of *E. coli* [56]. This discrepancy most likely derives from the difficulties in reconstituting *in vitro* a *bona fide* initiation complex reproducing the *in vivo* situation, particularly with a eubacterial RNA polymerase very different from NEP. Therefore, the structural motifs containing the transcription initiation sites are clearly distinct in the two viroids: terminal loops in ASBVd and double-stranded motifs in PLMVd.

### 4.2. Cleavage of oligomeric (+) and (−) strands is mediated by hammerhead ribozymes

Apart from the viroid discovery itself, the finding —first in ASBVd and then in the other members of the family *Avsunviroidae* [12,50]— that the strands of both polarities self-cleave through hammerhead ribozymes has attracted much attention on these small RNA replicons, which are now regarded as remnants of the RNA world postulated to have preceded the present world on Earth based on DNA and proteins [57]. Previous descriptions of the hammerheads found in viroids and in other small RNAs, and of the evidence supporting that they mediate self-cleavage *in vivo* of the multimeric viroid replicative intermediates wherein they are embedded, have been reviewed elsewhere [12,50]. Here we will focus on recent advances, which once again, have provided some surprises.

**Figure 4.**
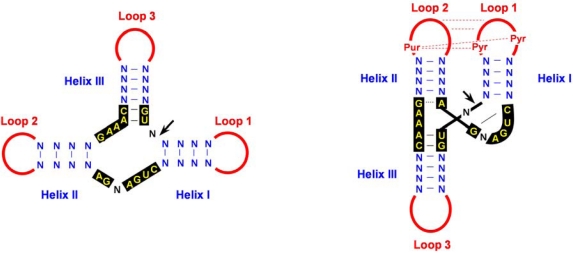
Hammerhead structures. **(A)** Schematic representation of a typical hammerhead structure as originally proposed [45]. Residues strictly or highly conserved in natural hammerhead structures are in yellow on a black background. Arrow marks the self-cleavage site, N indicates any residue and H any residue except G, and continuous and broken lines denote Watson-Crick and non-canonical base pairs, respectively. **(B)** Hammerhead structure represented according to X-ray crystallography and NMR data [60–62]. The proposed tertiary interaction between loops 1 and 2, which facilitates catalytic activity *in vivo,* is indicated with red broken lines. Pyr and Pur refer to pyrimidine and purine, respectively.

Hammerhead structures are formed by a central core of conserved sequences surrounded by three double-stranded stems (I, II and III) with loose sequence requirements and usually capped by short loops (1, 2 and 3). X-ray crystallography has unveiled that the actual conformation of these catalytic motifs does not resemble a hammerhead, as suggested by the initial bidimensional representation, but rather a Y wherein stems III and II are almost colinear (Figure 4). Data obtained with model hammerheads acting in *trans* (an artificial design with opened loops 1 and 2 that facilitates kinetic analysis in protein-free media) showed that efficient cleavage *in vitro* requires Mg^2+^ concentrations of 5–10 mM, an order of magnitude higher than that existing *in vivo*. However, re-examinations *in vitro* and *in vivo* have demonstrated that natural *cis*-acting hammerheads with intact loops 1 and 2 self-cleave much faster (retaining activity at 0.5-1 mM Mg^2+^), and that modifications of loops 1 and 2 cause a severe reduction of their catalytic activity [58,59], thus supporting that loop-loop tertiary interactions play a key role in the folding and catalytic activity of natural hammerheads. The recent X-ray crystal structures of two full-length natural hammerheads indeed show that the tertiary contacts, despite being distal from the active site, promote the adoption of a catalytically active conformation by the central core [60,61]. Moreover, applying a combination of NMR (nuclear magnetic resonance) spectroscopy, site-directed mutagenesis and kinetic and infectivity analyses to the (+) and (−) hammerheads of CChMVd has revealed that, in both hammerheads, loop 1 is a heptanucleotide hairpin loop containing an exposed U at its 5′ side and an extrahelical U at its 3′-side critical for the catalytic activity of the ribozyme *in vitro* and for viroid infectivity *in vivo*, whereas loop 2 has a key opened A at its 3′-side. These structural features promote a specific loop-loop interaction motif across the major groove, the structural features of which —base pairing between the 5′ pyrimidine of loop 1 and the 3′ purine of loop 2, and interaction of the extrahelical pyrimidine of loop 1 with loop 2 (Figure 4)— are likely shared by a significant fraction of natural hammerheads [62].

Two additional points regarding hammerhead function in the natural context deserve a comment. First, the loop-loop tertiary interactions could be further stabilized *in vivo* by chloroplastic proteins acting as RNA chaperones [63]. Further dissection of the processing requirements in the family *Avsunviroidae* may be tackled with a system based in transplastomic *Chlamydomonas reinhardtii* expressing (+) and (−) dimeric transcripts; despite the absence of viroid RNA-RNA transcription, this system can be used to identify the cellular factors facilitating cleavage (and ligation, see below) [64]. And second, the differential effects in co- and post-transcriptional self-cleavage of mutations affecting the trinucleotide preceding the self-cleavage site of (+) and (−) ELVd hammerheads suggest that: i) natural hammerheads have been evolutionarily selected to function co-transcriptionally, and ii) the trinucleotide AUC preceding the self-cleavage site is most likely excluded in the majority of natural hammerheads because it promotes the transient adoption of catalytically-inactive metastable structures during transcription [65]. Therefore, hammerhead-mediated catalysis appears closely associated with RNA folding taking place during transcription.

### 4.3. Ligation: an unsolved question with several possible alternatives

First, considering that the 5′-OH and 2′, 3′-cyclic phosphodiester termini resulting from hammerhead-mediated self-cleavage are those typically required by the wheat germ tRNA ligase, whose homologue from *A. thaliana* is the only plant RNA ligase well-characterized biochemically and genetically [66], and considering also its low substrate specificity, an enzyme of this class appears an excellent candidate for catalyzing RNA ligation in chloroplastic viroids. However, pre-tRNA splicing in yeast has been assumed to occur in the nucleus and, in consonance with this view, immunofluorescence and immune electron microscopy detected the tRNA ligase in this organelle [67]. This long-held paradigm has been questioned by the finding that yeast pre-tRNA splicing takes place in the cytoplasm [68]. Most importantly, in *A. thaliana* and *Oryza sativa* two of the splicing enzymes, the tRNA ligase and 2′-phosphotransferase (which catalyzes removal the 2′-phosphomonoester group generated by the tRNA ligase), have transit signals at their N-termini and are predominantly targeted to chloroplasts and proplastids, respectively [69]. Therefore, the plant tRNA ligase now fulfills all criteria for mediating circularization of members of the family *Avsunviroidae.* Moreover, in one viroid-like satellite RNA the junction resulting from ligation of the two termini generated by hammerhead-mediated self-cleavage contains the expected signature of a tRNA ligase: a 2′-phosphomonoester, 3′, 5′-phosphodiester group [70]. Encapsidation of this viroid-like satellite RNA by the coat protein of its helper virus might have precluded removal of the 2′-phosphomonoester. In this same direction we have observed in some PLMVd and CChMVd variants a deletion of the nucleotide immediately after the self-cleavage site that strongly reduces *in vitro* self-cleavage and infectivity. We suspect that this deletion may have been introduced during reverse transcription, because it was detected only in the plus-polarity strand, and that its ultimate cause could be the presence in a fraction of the viroid population of a 2′-phosphomonoester at the nucleotide preceding the self-cleavage/ligation site, which would lead to a jumping of the reverse transcriptase [71–73]. Another possibility is the 2′, 5′-phosphodiester linkage formed in the *in vitro* self-ligation of PLMVd, assuming that this atypical bond is indeed present *in vivo* (see below).

Second, the hammerhead ribozyme may mediate not only self-cleavage but also ligation generating a 3′, 5′-phosphodiester linkage. This is indeed the case for another small ribozyme, the hairpin ribozyme, which is found embedded in certain viroid-like satellite RNAs [74]. When compared with the hammerhead ribozyme, the RNA ligase activity of the hairpin ribozyme is much higher. However, recent data show that the ligase activity is considerably increased in hammerheads wherein the tertiary stabilizing interaction between loops 1 and 2 is preserved [75,76]. Therefore, it is still possible that replication of chloroplastic viroids might be an RNA-mediated mechanism only requesting a host RNA polymerase.

Third, the reaction could also be autocatalytic (self-ligation) but operating through a different pathway. This hypothesis is based on the observation that the monomeric linear PLMVd RNAs resulting from hammerhead-mediated self-cleavage can self-ligate *in vitro* mostly producing 2′, 5′-instead of the conventional 3′, 5′-phosphodiester bonds [77], and on the proposal that these atypical bonds exist in circular PLMVd RNAs isolated from infected tissue and impede their *in vitro* self-cleavage [78]. However, this may not well be the physiological mechanism because: (i) *in vitro* self-ligation of PLMVd demands concentrations of Mg^+2^ around 100 mM, which are much higher than those existing *in vivo*, ii) *in vitro* self-ligation has also been observed in PSTVd [23] and in HDV RNA, in this latter case also generating 2′, 5′-phosphodiester bonds [79], while neither PSTVd nor HDV RNA appear to follow this route *in vivo* [26,32,80], and iii) there are no previous reports on the existence of natural RNAs with 2′,5′-phosphodiester bonds serving as transcription templates; indeed, this atypical linkage blocks elongation catalyzed by the reverse transcriptase *in vitro* [77] and presumably by the NEP *in vivo*, while we have not observed prominent stops at the ligation site in our primer extension experiments using ASBVd circular forms isolated from infected tissue [81].

## 5. Concluding remarks and perspectives

The lack of protein-coding capacity of viroids entails that their replication mechanism is much more host-reliant than that of RNA viruses, which at least encode a subunit of the RNA-dependent RNA polymerase catalyzing initiation and elongation of viral strands. Moreover, viroids are able to redirect certain nuclear and chloroplastic RNA polymerases to transcribe RNA instead of their physiological DNA templates. Although the nature of the RNA polymerases involved in viroid replication appears well-established, they most likely act in concert with other host proteins that facilitate their template switching and processivity. New research avenues include identification of these proteins, as well as of the RNase mediating cleavage of oligomeric (+) replicative intermediates in the family *Pospiviroidae* and of the RNA ligases catalyzing circularization in both families (if this step is indeed an enzyme-mediated reaction in the family *Avsunviroidae*).

On the other hand, viroid replication may be closely linked to other biological features. Here are three illustrative examples. First, is viroid pathogenesis dependent or independent of replication? Although there is a report indicating that the ectopic expression of non-replicating viroid sequences incites typical symptoms [82], a recent work has failed to reproduce these results [83]. Therefore, symptoms may well be an effect of viroid replication and, more specifically, of detracting from their normal roles for some enzyme(s) of the RNA silencing pathways. Second, for the host range of members of the family *Avsunviroidae* —essentially limited to the natural hosts in which they were first reported [12,49], is it a consequence of restrictions in the access of these RNAs to the chloroplast or in their replication once they have been translocated into this organelle? And third, for the extremely high mutation rate of CChMVd, the highest reported for any biological entity [84], is it a result of being replicated by a proofreading-deficient chloroplastic DNA-dependent RNA polymerase redirected to using RNA? In other words, do other chloroplastic viroids have similar mutation rates as their rapid accumulation of sequence heterogeneity suggests? And what happens with nuclear viroids that are replicated by a different RNA polymerase and display a considerably lower sequence heterogeneity?
